# Air Pollution Emissions 2008–2018 from Australian Coal Mining: Implications for Public and Occupational Health

**DOI:** 10.3390/ijerph17051570

**Published:** 2020-02-29

**Authors:** Michael Hendryx, Mohammad Saidul Islam, Guang-Hui Dong, Gunther Paul

**Affiliations:** 1 Department of Environmental and Occupational Health, School of Public Health, Indiana University, Bloomington, IN 47405, USA; 2 School of Mechanical and Mechatronic Engineering, Faculty of Engineering and Information Technology, University of Technology Sydney, Ultimo, NSW 2007, Australia; MohammadSaidul.Islam@uts.edu.au; 3 Department of Environmental and Occupational Health, School of Public Health, Sun Yat-sen University, Guangzhou 510275, China; Donggh5@mail.sysu.edu.cn; 4 Australian Institute of Tropical Health and Medicine, James Cook University, Townsville, QLD 4810, Australia; Gunther.paul@jcu.edu.au

**Keywords:** air pollution, coal mining, Australia, public health, occupational exposure

## Abstract

Occupational exposure limits for respirable coal dust are based on exposure during working hours, but coal miners may experience additional community-based exposures during nonworking hours. We analyzed Australia National Pollutant Inventory (NPI) data for the years 2008–2018 to estimate air pollutants (metals, nitrogen oxides, particulate matter ≤ 10 micrometers (PM10) and ≤2.5 micrometers (PM2.5)) originating from coal mines. PM10 levels from community-based air monitors in Queensland and New South Wales were also compared between mining and nonmining communities. Results indicated that tons of coal mined increased over the study period, and that levels of particulate matter, metals, and nitrogen oxides increased significantly over time as well. Coal mines accounted for 42.1% of national PM10 air emissions from NPI sites. PM2.5 from coal mines accounted for 19.5% of the national total, metals for 12.1%, and nitrogen oxides for 10.1%. Coal mining occurred in 57 different post codes; the 20 coal-mining post codes with the highest PM10 emissions were home to 160,037 people. Emissions of all studied pollutants were significantly higher from coal mining sites than from other types of NPI sites. Results from community-based air monitoring stations indicated significantly higher population PM10 exposure in coal mining communities than in nonmining communities. The health of the public at large is impacted by coal mining, but to the extent that miners also live near coal mining operations, their total exposure is underestimated by consideration of exposure only during working hours.

## 1. Introduction

Coal mining is a hazardous occupation, with high risks for both accidents [1] and occupational diseases, especially respiratory disease [2,3], compared to other types of work. Occupational disease risks are not restricted to underground miners but extend to surface mining workers as well [4,5]. Surface mining workers are exposed to respirable dust and silica, and are at risk of coal workers’ pneumoconiosis [6]. 

In addition to occupational risks, coal mining also affects surrounding communities. Surface mining in particular contributes to local air pollution [7,8,9], with documented genotoxic effects [8] and increased risks for cancer, cardiovascular disease, and respiratory disease, among community populations [10,11,12,13]. A recent study in Australia reported that air quality as measured by sulfur dioxide (SO_2_), nitrogen oxides (NOx), or particulate matter with aerodynamic diameter ≤ 10 micrometers (PM10) or ≤2.5 micrometers (PM2.5) was significantly elevated in coal mining regions of New South Wales compared to other parts of the state [14]. 

Coal dust is a fine powder that forms during the mining process and from grinding and crushing of coal. The size distribution and chemical composition of coal dust particles are highly complex. The coal mining process generates both inhalable (<0.1 mm diameter) and respirable particles (<0.004 mm diameter). Inhalable particles are those that enter the nose or mouth, and respirable particles are the mass fraction of inhaled particles that penetrate the unciliated airways [15]. Coal dust contains organic maccerals and inorganic minerals (e.g., quartz silica, phyllosilicates, and sulfides) that could lead to respiratory cell damage [16]. Respirable coal dust particles can overcome the filtration system of the oral and nasal airways due to their small size, and can transport to the lower region of the airways [17]. Coal dust may contact the vessels of the alveolar airway, submucosa, and epithelium cells of the alveoli [18]. Respirable particles can potentially overcome the epithelium cells and capillaries and enter into the blood vessels of the respiratory system [19]. Respirable coal dust may promote inflammation of the alveolar epithelium cells and reduce mucociliary clearance [20]. Respirable coal mine particles influence overproduction of reactive oxygen species at the deeper airways, which eventually devastate the antioxidant system of the respiratory cell [15]. 

Long-term occupational exposure, and high concentration of coal dust and toxicity of respirable coal dust particles, leads to a variety of pulmonary diseases for coal mine workers. Coal workers’ pneumoconiosis (CWP) [16], silicosis [5], chronic obstructive pulmonary disease (COPD) [21], and mixed dust pneumoconiosis [5] are the most common respiratory diseases for coal mine workers. Silicosis is a dangerous respiratory disease mainly caused by respirable crystalline silica [22]. The published literature indicates that respirable crystal silicates are highly toxic and carcinogenic, and are responsible for causing cancer in lung tissues [23]. COPD and lung cancer have also been documented to occur in association with community, nonoccupational exposures to surface coal mining [11,13]. 

From 1 November 2018, the Queensland Occupational Exposure Limit (OEL) for respirable coal dust is 2.5 mg/m^3^ (<PM10 and median cut point PM4), measured as mean air concentrations over 8 h and across a defined number of workers, with proportional adjustments for longer shifts or more working hours per week [24]. Exposure standards for workers do not consider community exposures that may exist outside of working hours. To the extent that coal miners live in nearby communities, they may be at risk for exposure during nonworking hours. To more fully understand exposures that workers face, as well as to better characterize community level exposures that result from coal mining, consideration of pollutant emissions from mining sites that affect community populations should be investigated. We used National Pollutant Inventory (NPI) data to estimate air pollution emissions from coal mines that may impact surrounding communities, and evaluate trends over time in relation to changes in mining activity. 

## 2. Materials and Methods

### 2.1. Design

The study is a secondary analysis of existing data from the Australia National Pollutant Inventory (NPI) for the years 2008–2018, and data on coal production for the same years. Coal production data are from the Department of Industry, Innovation, and Science; production is measured as total raw black coal in million tons [25]. Secondary analysis of community-based air quality monitoring station exposure data for New South Wales and Queensland was also undertaken as described below.

### 2.2. Data

NPI data are from the Australian Government Department of the Environment and Energy [26]. The data provide the amount in kilograms (kg) of chemical emissions from thousands of individual sites or facilities in the country coded as to latitude and longitude, for each year 1998 to 2018. The current study examined emissions specifically from coal mining sites as identified by the Australian and New Zealand Standard Industrial Classification (ANZSIC) primary industry code in the NPI. PM2.5 was added to the NPI database beginning in the 2007–2008 reporting year (which we refer to as 2008), and the current study is therefore limited to the years 2008–2018. There are a total of 93 pollutants included in the NPI including emissions to air, water, and land. The current study was limited to air emissions that are predominant at coal mining sites, including PM10, PM2.5, nitrogen oxides (NOx), and metals. Metals included metals and metal compounds, including antimony, arsenic, beryllium, cadmium, chromium (VI), cobalt, copper, lead, manganese, mercury, nickel, selenium, and zinc. Air releases included point emissions, fugitive emissions, and total emissions. Fugitive emissions are releases not confined to a stack, duct, or vent, including equipment leaks, emissions from bulk handling or processing, windblown dust, and other industrial processes [17]. The current study used total air emissions, but at coal mining sites these are predominantly fugitive emissions. 

Emissions in the NPI database are measured via direct sampling or measurement, mass balance calculations, fuel analysis, or other engineering calculations, or via production-based emissions factors [27]. Production-based emission factors constituted about 99% of estimation methods used for fugitive air emissions at coal mines. Calculation of production-based emission factors for mining as described by the NPI [28] included a set of equations that considered activity rates, operating hours, emission factors for specific pollutants depending on the type of mining operation (draglines, shovels, truck haulage, etc.), and pollution control processes (e.g., water sprays). 

In a subsequent limited case-study exposure analysis, PM10 levels measured in community-based air monitoring stations were compared between mining and nonmining locations. These data were limited to daily mean PM10 (µg/m^3^) in the year 2017 and to the two Australian states of New South Wales and Queensland based on data available to the authors. Most Australian coal mining occurs in these two states. Nine observations were missing from 2017 data for Queensland and were replaced with 2016 or 2018 data. Data from New South Wales (NSW) were obtained from the NSW Office of Environment and Heritage Air Monitoring Network [29]. Data for Queensland were queried from a Queensland Government web interface [30]. Community monitoring stations were categorized into three groups: those located in communities within less than 100 km of coal mining, those in nonmining urban areas, and those in nonmining rural areas. Annual hourly data for each monitoring stationed was averaged, and all error data were omitted. 

### 2.3. Analysis

Analyses included descriptive summaries of emissions for metals, particulate matter (PM2.5 and PM10), and NOx, overall and by year. Correlational trends for emissions were monitored over time corresponding to data on mining production. We also estimated population exposures for coal mining sites with the highest amounts of emissions, and estimated the percent of national emissions that originated from coal mining. We used two-tailed t-tests to determine whether levels of emissions were significantly different between coal mining sites and all other NPI sites; we corrected where necessary for unequal variances by using Satterthwaite t-values. 

PM10 levels from the community air monitoring stations were statistically compared between the three groups (mining, nonmining urban, and nonmining rural) using general linear models. All analyses were conducted using SAS software version 9.4 (Cary, NC, USA).

## 3. Results

The total amount of air emissions in kilograms from coal mines in Australia for the selected emission types is provided in Table 1. The number of observations refers to mine sites times years. Metals emissions were dominated by manganese, followed by zinc, nickel, and copper. PM10 emissions were substantially higher than any other single type. 

**Figure 1:**
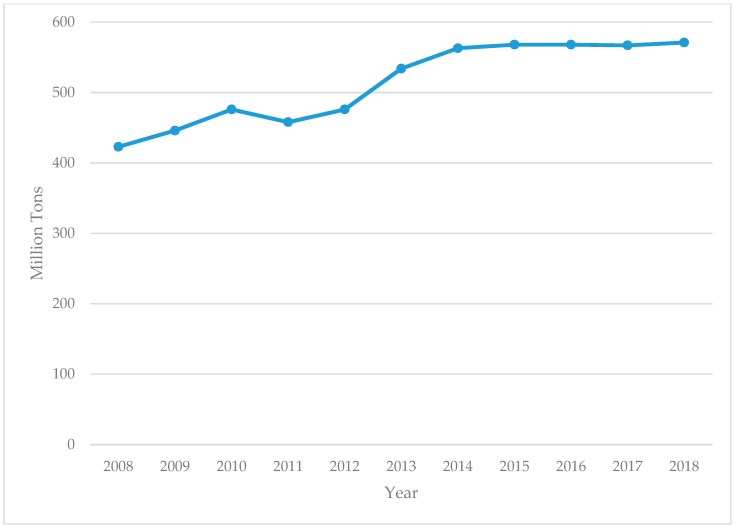
Coal production in million tons, 2008–2018.

Between the years 2008–2018 there was an increase in coal production in Australia (Figure 1). Coal production increased from 423 million tons in 2008 to 571 million tons in 2018, although production was essentially flat in the later years 2014–2018. Using 2008 as baseline, coal production in Australia increased by 35% by 2018.

**Figure 2:**
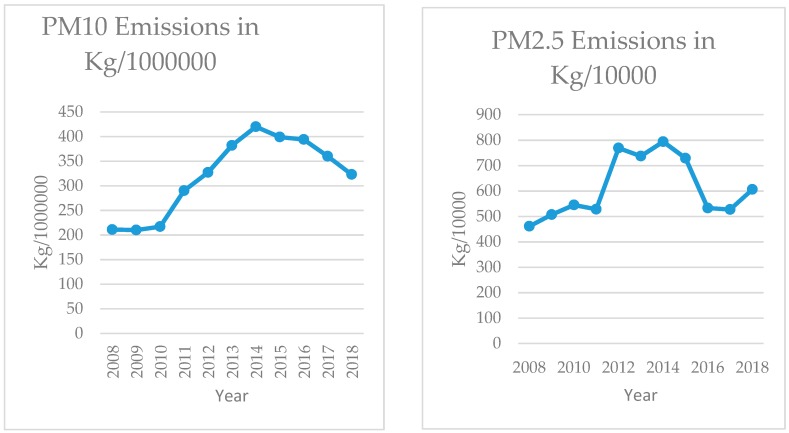

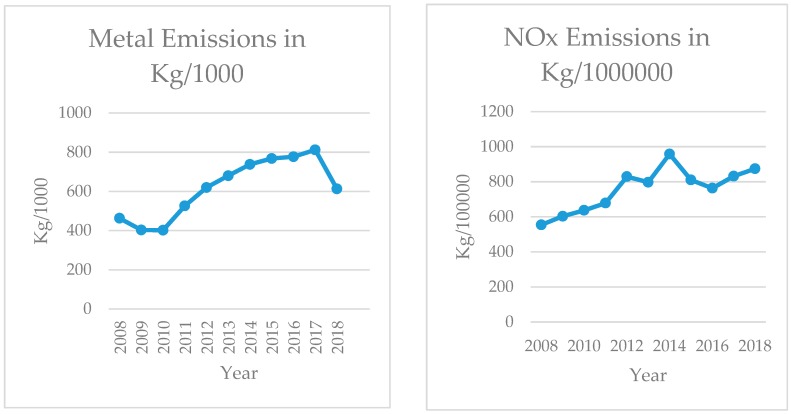
Air pollution emissions from coal mining sites, 2008–2018.

Because pollution estimates are based on production-based emission factors, it is not surprising that levels of pollution emissions also increased between 2008 and 2018. Figure 2 shows emission quantities by year for PM10, PM2.5, metals, and NOx. Emissions from all pollutants increased over the whole period, with highest levels generally in the middle period of the study interval (approximately 2012–2015) followed by reduced amounts in later years. For three of the four emission measures, the highest emissions were reported for 2014, corresponding to the year when coal production essentially peaked, then were reported to decline although production remained flat.

**Figure 3:**
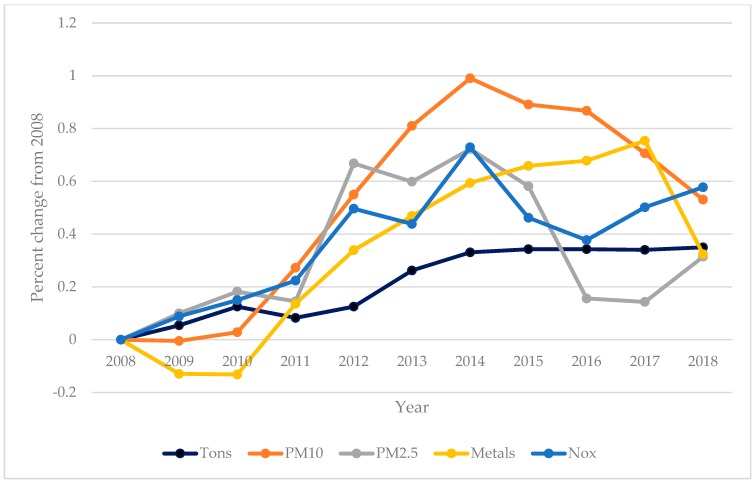
Percent change from 2008 in tons of coal production and in pollution emissions.

Figure 3 rescales the changes in tons of coal production and pollution emissions as a percent change from the 2008 baseline. This was done to set production and emissions to the same scale for comparative purposes. As noted above, coal production increased by 35% from the first to the last study year. Pollution emissions as percentages increased by greater amounts; PM10 increased by almost 100% by 2014 before falling to an increase of 53% by 2018. PM2.5, metals, and NOx also increased relatively more than coal production, especially in middle years. Statistical tests for annual trends indicated that increases in pollution emissions and in coal production were all significantly greater than zero.

We next identified the post codes corresponding to coal mining sites in Australia. Coal mining took place across 57 different post codes in Australia. Table 2 lists the 20 post codes with the highest PM10 emissions from coal mines. The Table includes total kg of emissions and the population of the post code according to the 2016 Australian Bureau of Statistics [31]. All of the sites were located in Queensland or New South Wales with the exception of one post code in Western Australia. The Table shows that most mining locations were in rural areas with small populations, but that 160,037 people lived in the post codes representing the top 20 PM10 emissions areas.

Table 3 shows the percent of national air emissions from the NPI that originated from coal mines. Coal mines accounted for 42.1% of national PM10 air emissions from sites included in the NPI. PM2.5 from coal mines accounted for 19.5% of the total, metals for 12.1%, and NOx for 10.1%.

Table 4 indicates that PM10, PM2.5, NOx, and metals emissions were all significantly higher at coal mining sites than at other combined types of NPI sites.

Results from the community-based air monitoring stations showed that PM10 levels were significantly higher in the communities near mining sites, which are predominantly located in rural areas, than in nonmining urban communities or nonmining rural communities (Table 5).

## 4. Discussion

The results of the study indicate that air pollution originating from coal mining sites in Australia increased over the years 2008–2018 corresponding to increases in mining production. Emissions of NOx, particulate matter, and metals were significantly higher from coal mining sites than from other types of NPI sites. This air pollution affects community populations in the vicinity of mining sites, as estimated by emissions data from the NPI in the current study; by the results of our limited analysis of PM10 levels from community monitors; and as estimated by data from stationary community-based air quality monitors as previously reported [14]. PM10 emissions from coal mining sites are particularly noteworthy, accounting for over 42% of national PM10 emissions in the NPI database, but pollution from PM2.5, metals, and nitrogen oxides are also significant. These air pollutants have been well established by prior literature to contribute to premature population mortality and morbidity [32,33,34,35].

Coal miners face occupational exposures, which were found to be 2.1 mg/m^3^ in average in Queensland underground coal mining in 2002 [36], but investigations into total exposures for miners should appreciate that communities are also exposed to pollutants and miners who live in these communities are exposed even when not working. Consideration of occupational exposure standards may benefit from incorporating both occupational and nonoccupational estimates of exposures among miners.

In addition to miners, air pollution from mining activities, including extraction, processing, and bulk transport, impacts the health of the community at large [10,37,38]. Our analysis confirmed that PM10 exposure levels were significantly elevated in mining communities relative to urban or rural nonmining communities. These elevated levels are present despite the relatively small populations in mining districts, at amounts higher than found in larger urban settings. Vulnerable populations (e.g., children, pregnant women, elderly people, or those with pre-existing conditions) may be at most risk from mining related pollutants [10,39,40,41].

Trends over time indicate that pollutant emissions and mining activity were related, as would be expected based on estimating emissions from production-based equations. However, correspondence was not exact. Reported emissions during the middle years of the observation period increased disproportionately to tons of coal mined, and then declined in later years as production remained flat. Reasons for this are unclear, but may reflect either variation in production intensity required to extract a given quantity of coal, or error in estimation practices. The sharp drop in the metals emissions estimate for 2018, for example, seems difficult to reconcile with tons of coal mined for that year. A limited study of lead (Pb) emissions data from the NPI concluded that lead emissions were underreported [42], and it is possible that underreporting took place for data used in the current study as well. To the extent that underreporting bias in emissions reporting may exist, bearing in mind we still detected associations between emissions and mining levels, associations between mining and PM10 community exposures, and substantial contributions of mining to national emission levels, the estimated magnitude of these mining emissions contributions may be conservative.

In addition to uncertainty about production-based estimates, a number of other study limitations should be recognized. NPI data are limited to stationary sites, and do not include mobile sources. The study examined 13 metals, nitrogen oxides, PM2.5, and PM10; there are, however, 93 total pollutants included in the NPI and coal mines that have emissions of 44 of these pollutants, including volatile organic compounds, polycyclic aromatic hydrocarbons, carbon monoxide, and many others [26]. Total air pollution emissions from coal mines represent a complex mixture of coexposures, which may have larger implications for both occupational and community health. Air monitoring stations in coal mining areas are scarce; in Queensland and New South Wales, for example, there are 19 such monitors compared to 45 in nonmining urban areas. No direct health assessments were made in this study, although considerable additional evidence indicates that coal mining adversely impacts public health [10,43].

## 5. Conclusions

In conclusion, the current study documents substantial air pollution emissions from coal mines in Australia, as well as higher levels of particulate matter measured in community air monitoring stations in mining versus nonmining communities. Emissions and subsequent exposures likely have impacts on the health of surrounding communities. Future research may more directly test associations between emissions from mining sites and community exposures, following approaches used in source apportionment studies that have linked air pollution to coal-based power generation [44]. As suggested by the current study and much prior research, the health of the public at large is influenced by coal mining, but to the extent that miners also live near coal mining operations, their total exposure is underestimated by consideration only of exposure during working hours. Regulatory exposure limits for community populations and those for occupational populations differ in limits and exposure period, making it difficult to estimate what total exposure limits may be suitable for occupational populations that also experience community exposures, but efforts to develop more comprehensive exposure guidelines would be beneficial and may result in a lowering of occupational exposure limits.

## Figures and Tables

**Table 1:** Air emissions from coal mines in kilograms, 2008–2018.

Emission	N of Observations	Total Amount in kg
Antimony and compounds	741	7402.5
Arsenic and compounds	985	44,023.9
Beryllium and compounds	902	12,776.9
Cadmium and compounds	866	2299.9
Chromium (VI) and compounds	107	1565.7
Cobalt and compounds	967	74,179.6
Copper and compounds	1089	241,099.8
Lead and compounds	1102	153,289.6
Manganese and compounds	1161	4,844,927.9
Mercury and compounds	1114	1393.3
Nickel and compounds	1090	281,789.1
Selenium and compounds	667	9982.4
Zinc and compounds	1117	1,127,249.7
Total Metals	11,908	6,801,980.3
PM2.5	1071	67,361,544.1
PM10	1082	3,534,238,028.0
NOx	1074	833,567,946.0

**Table 2:** Post codes and populations corresponding to the 20 locations with highest PM10 emissions from coal mining sites (post codes starting with: 4…: Qld; 2…: NSW; 6…: WA).

Post Code	PM10 Emissions (kg)	Population (2016)
2330	517,080,520	20,350
4744	487,372,265	8735
4742	393,371,886	1140
4717	320,200,354	4749
4746	234,138,583	1935
4745	224,807,477	2991
2333	195,740,657	13,647
4718	152,534,402	2375
4720	104,375,624	14,394
4715	84,736,324	7127
2382	83,557,341	1319
4743	81,607,887	620
4702	75,395,426	28,609
4721	71,774,189	3717
2850	63,454,014	17,644
4741	52,933,785	7079
4804	47,928,470	1,730
4401	45,444,892	5719
6225	41,986,061	9105
4615	28,489,238	7052

**Table 3:** Coal mining air emissions as a percent of total air emissions, Australia, 2008–2018.

Emission	Percent of National Air Emissions from Coal Mines	Total National Air Emissions (kg)
Metals	12.1%	56,120,926.2
NOx	10.1%	8,267,252,710
PM2.5	19.5%	344,873,749
PM10	42.1%	8,404,128,401

**Table 4:** Mean (standard error) air emissions in kg from coal mines compared to other NPI sites, Australia, 2008–2018.

Emission	Coal Mines	Other NPI Sites	*p* < *
Metals	571.2 (24.1)	485.6 (36.4)	0.05
NOx	776,134 (30,071)	358,423 (14,890)	0.0001
PM2.5	62,896 (2626)	13,943 (597)	0.0001
PM10	3,266,394 (136,801)	235,807 (9354)	0.0001

* *p* values based on two-tailed t-tests with Satterthwaite correction for unequal variances where appropriate.

**Table 5:** Mean and standard deviation PM10 levels (µg/m^3^) for 2017 at community air monitoring stations in Queensland and New South Wales.

Group	N of Monitoring Sites	Mean	Standard Deviation	*p* < *
Mining	19	20.71	3.79	<0.02
Nonmining urban	45	17.36	4.53	
Nonmining rural	12	16.04	6.26	

* *p* value from general linear model (F = 4.67, df = 2, 73, *p* < 0.02).
